# Paternal Cocaine Exposure and Its Testicular Legacy: Epigenetic, Physiological, and Intergenerational Consequences

**DOI:** 10.3390/biology14081072

**Published:** 2025-08-18

**Authors:** Candela R. González, Betina González

**Affiliations:** Instituto de Investigaciones Farmacológicas, Universidad de Buenos Aires–Consejo Nacional de Investigaciones Científicas y Técnicas, Ciudad Autónoma de Buenos Aires, Junín 956, piso 5, Buenos Aires C1113, Argentina

**Keywords:** cocaine, male reproduction, spermatogenesis, catecholamines, sperm epigenome, epigenetic inheritance

## Abstract

Cocaine use remains a major public health concern, with rising global prevalence and a well-established profile of neurotoxicity and addictive potential. While the central nervous system has been the primary focus of cocaine research, emerging evidence indicates that cocaine also disrupts male reproductive physiology. In the testis, cocaine alters the endocrine microenvironment, induces cell-specific damage, and disrupts spermatogenesis. Cocaine also interferes with epigenetic programming in germ cells and mature sperm, potentially leading to heritable epimutations. Epidemiology data reveal that approximately two-thirds of regular cocaine users are males of reproductive age, and preclinical models have documented numerous behavioral and molecular alterations in their offspring, often linked to paternal cocaine exposure—such as increased drug resistance or vulnerability, altered anxiety-like behavior, impaired learning/memory, disrupted social behaviors, and shifts in neural circuitry and gene expression in reward-related brain regions. This review aims to integrate findings from studies that have independently examined testicular dysfunction, germline epigenetic reprogramming, and offspring outcomes, offering a unified perspective on their potential interconnections and highlighting future directions for research in the field of epigenetic inheritance.

## Simple Summary

This review integrates current evidence on the effects of paternal cocaine exposure on the testis and its potential epigenetic transmission to the next generation. While the neurotoxic profile of cocaine is well established, emerging data highlight its capacity to disrupt the male reproductive axis, impairing hormonal balance, altering the testicular microenvironment, and inducing cell-specific damage. We also explore how cocaine modulates testicular catecholaminergic signaling—a previously underrecognized local pathway—and its potential role in germline reprogramming. Notably, exposure during spermatogenesis can interfere with key epigenetic mechanisms, including DNA methylation, histone modifications, and non-coding RNA expression, leading to stable changes in the sperm epigenome. These germline alterations may escape embryonic reprogramming and contribute to phenotypic and molecular abnormalities in the offspring. By compiling data from toxicology, neuroendocrinology, and germline epigenetics, this review offers a unified framework that brings together previously fragmented evidence. This review emphasizes the need for translational research to assess the reproductive and epigenetic risks associated with cocaine use in males of reproductive age.

## 1. Introduction

Addictive substance use is a growing public health concern, not only because of its immediate toxicological effects, but also because of its long-term impact on reproductive health and potential consequences for future generations. Among recreational psychostimulant drugs, cocaine stands out as a potent neuroactive and addictive compound that exerts systemic effects far beyond the central nervous system (CNS). Although the brain and the testis appear to be very different organs, research has revealed unexpected molecular, genetic, and functional similarities between them [1] (Gonzalez 2021). Both are immune-privileged sites due to the presence of specialized blood barriers, and they exhibit remarkably high transcriptional complexity [2]. These organs express a wide array of genes and splicing variants involved in cell-to-cell communication, vesicle trafficking, cytoskeleton dynamics, energy metabolism, epigenetic regulation, and cellular differentiation [2,3]. However, while cocaine neurotoxicity has been extensively studied, its impact on the testis has only recently begun to be elucidated. Mounting evidence from preclinical models suggests that cocaine disrupts spermatogenesis, alters testicular homeostasis, and induces molecular stress pathways in both somatic and male germ cells [1]. More critically, cocaine exposure during spermatogenesis has been shown to trigger epigenetic alterations in male germ cells, including changes in DNA methylation, histone modifications, and non-coding RNA profiles—modifications that can be retained in mature sperm. These epimutations not only compromise sperm quality but may also be transmitted to the offspring, affecting early embryonic development and long-term phenotype [4]. In this review, we aim to integrate evidence from testicular physiology, epigenetic regulation, and transgenerational inheritance to highlight emerging concepts and identify critical gaps for future research.

## 2. Cocaine as a Global Health Issue: Epidemiology and Neurobiological Mechanisms

According to the 2023 Global Report on Cocaine (UNODC), cocaine consumption has exhibited a sustained global increase over the past 15 years, driven not only by population growth but also by a rising prevalence of use across multiple regions [5]. (United Nations Office on Drugs and Crime (2023). Global Report on Cocaine 2023. Viena, Austria: UNODC). Established markets, including North America, Western and Central Europe, and parts of Australia, have shown an intensification in consumption patterns, with an increasing number of individuals engaging in frequent use. Epidemiological data from the UNODC indicate that two-thirds of regular cocaine users—about 22 million people worldwide—are males of reproductive age. This pattern is further illustrated by data from Brazil, where the prevalence of regular cocaine use peaks in males aged 18–30, underscoring the vulnerability of this reproductive-age group. Although female cocaine use has also increased notably in some regions, the UNODC reports a narrowing gender gap in cocaine consumption.

As global cocaine use continues to rise, its broad neurobiological and systemic effects warrant increasing attention. Cocaine is a potent psychostimulant that blocks the reuptake of the monoamines dopamine (DA), norepinephrine (NE), and serotonin (5-HT) by inhibiting their respective transporters—DAT, NET, and SERT—leading to sustained monoaminergic signaling in both the central nervous system (CNS) and peripheral tissues including the heart, vasculature, lungs and gut [6,7]. In particular, the impact of central DAT inhibition and increased DA neurotransmission in the reward circuitry has been widely investigated in cocaine addicts and animal models. The DA hypothesis posits that addiction is rooted in compromised dopaminergic systems, where overstimulation and downregulation of D1-like and D2-like (DRD1 and DRD2) DA receptors affect brain regions that are critical for reward processing, motivation, decision-making and habit formation [8,9,10,11]. The reward circuitry is characterized by the unique expression of DAT, which is considered to have evolved specifically to fulfill specialized roles in dopaminergic neurotransmission. Unlike peripheral tissues, where DA reuptake is primarily mediated by NET and other monoamine transporters such as organic cation transporters (OCTs) and plasma membrane monoamine transporter (PMAT), the DAT is selectively expressed in dopaminergic neurons within key brain regions including the striatum, nucleus accumbens (NAcc), and prefrontal cortex [12,13]. In these regions, DAT tightly regulates synaptic DA dynamics, which are essential for reward processing and motor control [12,13]. However, central dopaminergic mechanisms alone cannot fully explain cocaine rewarding and reinforcing effects, and the noradrenergic system also plays a key role, as these two catecholamines are closely related to the behavioral and systemic responses induced by stressors [14]. Catecholamine-secreting cells convert tyrosine to L-DOPA through the rate-limiting enzyme tyrosine hydroxylase (TH), and then to DA through decarboxylation by Dopa Decarboxylase (DDC). Depending on the cell type, DA may be further converted to NE by dopamine beta-hydroxylase (DBH) and epinephrine [1,15]. NET has comparable affinity for DA and NE, and NET inhibition by cocaine elevates both NE and DA levels [16]. Moreover, DA has higher affinity for NET than for DAT [17], and brain regions with sparse DAT expression seem to use NET for DA reuptake [18]. NET blockade thus enhances synaptic concentrations of both catecholamines, highlighting the functional coupling of the DA and NE systems [19]. This is particularly relevant in peripheral tissues, where NET is expressed in the sympathetic nerve terminals, adrenal medulla, myocardium, and vasculature, and NET inhibition leads to sympathetic overdrive, vasoconstriction, hypertension, and cardiotoxicity [20]. Additionally, cocaine also blocks SERT elevating extracellular 5-HT levels, with significant consequences in both CNS and peripheral tissues. Increased cocaine-induced serotonergic tone has been implicated in mood changes, vascular responses, immune modulation, and gastrointestinal alterations [21,22,23].

Beyond monoamine transporter inhibition, cocaine activates sigma-1 receptors (S1Rs), which are expressed in reward-related brain regions and, upon translocation, are physically associated with D1-like and D2-like DA receptors (DRD1 and DRD2) [24]. S1Rs receptors also exert modulatory effects on voltage- and ligand-gated ion channels, particularly on NMDA glutamate receptors, and increase mitochondrial calcium and reactive oxygen species (ROS) [25,26]. Consistently, cocaine acts as a strong disruptor of cellular redox homeostasis, promoting excessive ROS production, altering mitochondrial membrane potential, and impairing autophagy and mitophagy, which further promotes oxidative stress and inflammation [27,28]. Finally, cocaine also blocks voltage-gated sodium channels, producing its well-known local anesthetic effects [29,30], and dampening neural excitability, with an impact on sensory, motor, and cortical signaling [31].

These aforementioned cocaine-induced alterations underlie the emergence of compulsive drug-taking and are linked to cognitive impairments, mood disorders, autonomic dysfunction and cell toxicity, affecting multiple organs—including the heart, brain, liver, and kidneys—through complex toxic mechanisms. Furthermore, the increasing prevalence of cocaine use among males of reproductive age raises additional concerns regarding its potential effects on the germline. Given the well-established impact of cocaine on neuroendocrine and systemic pathways, growing evidence suggests that these alterations may reach the male reproductive system and induce epigenetic modifications in germ cells, potentially contributing to intergenerational transmission of drug-induced phenotypes. However, epidemiological studies in humans addressing the phenotypic consequences of paternal cocaine consumption on offspring are still lacking. Moreover, although the systemic effects of cocaine have been extensively studied, its impact on testicular physiology remains less thoroughly characterized.

## 3. Cocaine and Male Reproductive Physiology: From Hormonal Disruption to Cellular Effects

While several causes of male infertility are well-established—such as age, medications, environmental toxins, genetic factors, and endocrine disruptors [32,33,34]—the reproductive impact of lifestyle and behavioral factors, particularly cocaine use, has received comparatively less attention. Clinical studies suggest that cocaine can impair sexual function—causing erectile dysfunction and ejaculatory disorders—in humans [35], and can negatively affect sperm parameters in both humans and rodents [36,37]. Although the number of studies addressing cocaine’s effects in humans remains limited, early clinical evidence points to a significant association between cocaine use and impaired sperm quality. For instance, an early study by Bracken et al. found that men with sperm counts below 20 million/mL were twice as likely to have used cocaine in the previous two years compared to non-users [36]. Additional clinical evidence further supports that cocaine use decreased sperm motility, increased morphological abnormalities, and overall compromised sperm quality [36,38]. Moreover, high concentrations of cocaine have been shown to bind selectively to human sperm, reinforcing the hypothesis that spermatozoa may serve as vectors for delivering the drug to the oocyte [39].

In line with clinical observations, studies in animal models have shown that cocaine affect multiple levels of the male reproductive system, including hormonal regulation and testicular architecture, with direct consequences for spermatogenesis. Notably, following the administration of radiolabeled cocaine, the testis exhibits the second-highest drug accumulation after the brain [40]. Although specific binding sites for cocaine have been identified in testicular tissue [41], the precise mechanisms by which cocaine disrupts testicular physiology remain only partially elucidated.

### 3.1. Neuroendocrine Disruption and Hormonal Imbalance

Cocaine exposure interferes with endocrine regulation in males, primarily through disruption of the hypothalamic–pituitary–gonadal (HPG) axis. Several experimental studies in human trials have reported acute elevations in luteinizing hormone (LH) levels without corresponding increases in serum testosterone, suggesting a possible uncoupling of hypothalamic–pituitary signaling from testicular steroidogenesis [42]. In particular, chronic exposure—both in animal models and in cocaine-dependent men—has been consistently associated with reduced circulating testosterone concentrations [31,43,44]. Further declines have been observed in animals following prolonged or binge use [45,46,47], supporting the notion of cumulative endocrine disruption. In addition to testosterone, cocaine affects other key reproductive hormones. Acute administration has been shown to suppress prolactin (PRL) secretion in men, with levels remaining significantly below baseline [39]. This inhibitory effect on PRL may reflect dopaminergic modulation at the hypothalamic or pituitary level [48]. Furthermore, cocaine has been associated with decreased estradiol (E2) levels in rodents, a hormone known to play critical roles in spermatogenesis, particularly in germ cell maturation [49]. As a consequence, an increased testosterone-to-estradiol (T/E2) ratio has been observed, which may impair proper sperm development [49].

One of the most relevant effects of cocaine is the activation of the hypothalamic–pituitary–adrenal (HPA) axis in both humans and rodents, contributing to stress hormonal dysregulation that may promote drug-seeking behavior and relapse. In healthy men, acute cocaine administration increases plasma cortisol levels and disrupts the normal circadian decline of this hormone [50]. In cocaine-dependent subjects, stress-induced elevations in ACTH and cortisol have been linked to greater drug intake during relapse episodes, although not to the time to relapse onset [51]. Additional studies in animal models have shown that cocaine enhances the amplitude of ACTH and cortisol secretory pulses, confirming HPA axis activation [52]. In rodents, both acute and repeated exposure increase circulating corticosterone levels and amplify stress responses, likely via corticotropin-releasing hormone (CRH) signaling [53]. These findings support the idea that cocaine-induced sensitization of the HPA axis, particularly under stress, may compromise neuroendocrine homeostasis and heighten vulnerability to compulsive use.

### 3.2. Wired for Damage: Local Mechanisms of Cocaine Toxicity in the Testis

Despite extensive research on the central and systemic effects of cocaine, its impact on reproductive organs—particularly the testis—remains poorly understood, as only a limited number of studies have addressed its consequences on gonadal function. However, chronic cocaine exposure has been shown to disrupt testicular homeostasis through both neuroendocrine and local molecular mechanisms, potentially compromising spermatogenesis. In this context, a local catecholaminergic system has been identified in the testis, suggesting that this organ could be directly influenced by cocaine-induced dysregulation of stress-related signaling pathways.

**Figure 1:**
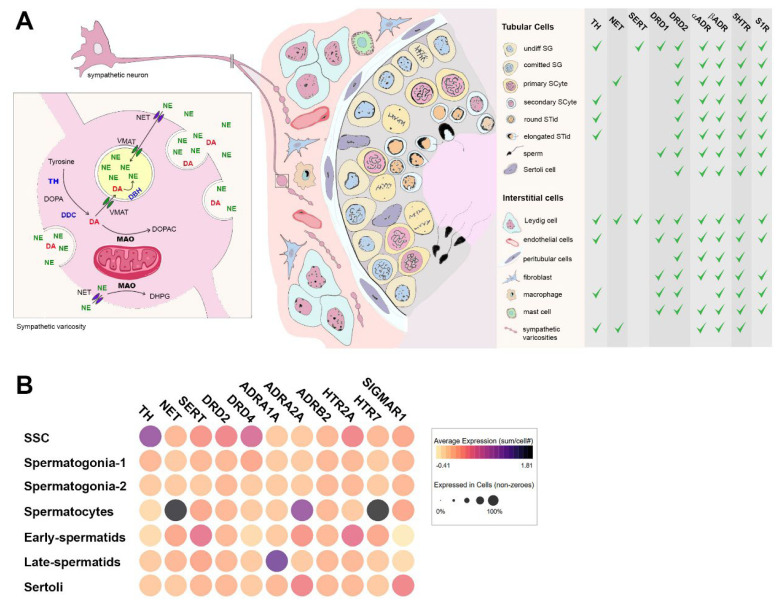
Testicular catecholaminergic circuit and local targets for cocaine response. (**A**) Schematic representation of the catecholaminergic components in the mouse testis. The figure depicts extrinsic sympathetic innervation and intrinsic catecholamine-expressing cells, which are located in the interstitial and tubular compartments. The extrinsic sympathetic innervation is characterized by bead-like axon terminals named “varicosities”, where catecholamine synthesis occurs locally. Local cells express catecholamine-synthesizing enzymes TH, DDC, and DBH, and monoamine transporters NET and SERT. Dopamine receptors (DRDs), adrenoreceptors (αADR and βADR), serotonergic 5HTR, and sigma S1Rs receptors are present in interstitial and tubular cell populations. (**B**) Expression of the main components of the monoamine systems’ human cell-specific single-cell RNA-seq data in the Human Testis integrated dataset, available at UCSC cell browser portal (https://testis.cells.ucsc.edu/, accessed on 15 May 2025). These local monoaminergic systems may represent a functional interface through which systemic cocaine exposure alters testicular signaling. Overall, the figure shows that cocaine acts on several testicular cell populations via both direct actions on monoamine transporters and sigma-1 receptors, and indirect actions that enhance monoaminergic tone through sympathetic and local mechanisms.

In the testis, cocaine targets are present in both the interstitial and tubular compartments, which express direct targets such as NET and SERT, as well as several DRDs, adrenergic receptors (ADRs), serotonin receptors (5-HTRs), and S1Rs (Figure 1A). The main structural and molecular components of the testicular catecholaminergic system are composed of the extrinsic sympathetic innervation, and intrinsic catecholamine-synthetizing interstitial and tubular cells. Sympathetic terminals are localized in the interstitial compartment and exhibit bead-like swellings along the axons, known as varicosities, where DA and NE are synthesized and released [1]. NET inhibition by sympathomimetic drugs like cocaine leads to increased catecholamine release, which in turn triggers a rise in glucocorticoid levels that further amplifies catecholaminergic signaling by upregulating TH expression in local cells [1,54,55]. In this sense, the testes of both humans and mice contain several cell populations that exhibit catecholamine synthesis capacity. The Leydig cells express TH, DDC and DBH, and they also show low levels of NET protein [56,57,58]. Other interstitial cells such as macrophages and endothelial cells also express TH, DDC, and DBH, particularly under stress conditions [15,59,60]. Neuron-like cells expressing TH and DAT have also been identified in the interstitial compartment of the human testis [61,62]. Interestingly, the tubular compartment also expresses catecholamine-synthesizing enzymes and NET. TH has been detected in undifferentiated spermatogonia and post-meiotic mouse germ cells [57], while high NET protein levels have been reported in human pachytene spermatocytes [58]. In line with this, DA immunoreactivity has been observed in both interstitial and tubular compartments [63], and increased testicular TH expression was found after chronic cocaine exposure in animal models [57].

Several dopaminergic and adrenergic receptors are specifically distributed across all cell populations that comprise the testis, being potentially responsive to both locally produced and bloodstream-circulating catecholamines. Activating DRD1 and inhibitory DRD2 are found in human and mouse Leydig cells [1,58]. DRD1 is also detected in mouse spermatogonia adjacent to the basal lamina, while DRD2 receptor is expressed in pre- and post-meiotic germ cells as well as Sertoli cells [1,57]. Consistently, we have shown that chronic cocaine exposure downregulates DRD1 and DRD2 expression in the mouse testis, mirroring the transcriptional alterations reported in the CNS [57,64]. Transcriptomic datasets from human and mouse testis also show that α1-ADR, α2-ADR, and β-ADR are expressed in male germ cells, with stage-specific expression patterns throughout spermatogenesis (Figure 1A) [1]. Other putative sites of cocaine actions in the testis are SERT and S1R. SERT is highly expressed in Leydig cells, and 5-HTR are widespread across interstitial and tubular cells [15]. This suggests the existence of a local serotonergic system that could also be modulated by cocaine, potentially impacting processes like testosterone production, spermatogenesis, and testicular blood flow [1,15]. As for S1Rs, they were mostly located in the tubular compartment, and also in Leydig cells in both human and mouse testis (Figure 1A) [58,65], but their role in the testis has not been explored. The established roles of S1Rs in oxidative stress and autophagy, as well as neurotransmitter and voltage channels modulation, makes it an interesting target to further evaluate cocaine toxic mechanisms in the testis.

Altogether, these findings support the notion that the testis is wired with a complex network of stress-responsive signaling pathways, which may represent critical molecular targets of cocaine and contribute to its local toxic effects.

### 3.3. Somatic and Germ Cell Vulnerability

The cellular impact of cocaine in the testis cannot be fully understood without considering its ability to activate convergent stress pathways that are also engaged in the CNS. As described above, cocaine enhances catecholaminergic signaling, disrupts mitochondrial function, impairs mitophagy, and promotes oxidative stress in multiple organs. These molecular events are not restricted to the brain, and growing evidence shows that similar alterations occur in testicular cells. For instance, increased ROS production, mitochondrial swelling, and increased autophagy markers have been consistently observed in somatic and germ cells after cocaine treatment [57]. Notably, elevated DA levels can contribute to oxidative stress, as its metabolism by monoamine oxidase (MAO) generates H_2_O_2_, and DA can also auto-oxidize to produce reactive quinones and superoxide [66,67]. These findings suggest that testicular cell populations are not merely passive targets but active responders to cocaine-induced stress.

Leydig cells undergo significant damage following chronic cocaine exposure. Crack-cocaine inhalation significantly increases apoptosis of Leydig cells in adult mice [68]. Given the role of catecholamines in modulating steroidogenesis [69,70], it is plausible that local dopaminergic imbalance induced by cocaine contributes to impaired androgen production. On the other hand, Sertoli cells are also targeted by cocaine. A marked decrease in Sertoli cell number has been reported in both young and adult mice following chronic crack-cocaine exposure, with more severe effects observed in younger animals [68]. This suggests that early exposure may disrupt Sertoli cell development, possibly via increased apoptosis or impaired proliferation. In vitro studies in rat Sertoli cells showed that cocaine decreases the secretion of transferrin and androgen-binding protein—two key factors for germ cell maturation [71]. Additionally, ultrastructural damage, including vacuolization and lipid accumulation, has been reported [72], and this is indicative of cocaine-induced metabolic stress.

Spermatogenic cells are particularly vulnerable to cocaine. In young mice, cocaine administration leads to an accumulation of round spermatids and a reduction in elongated spermatids, suggesting disruption of spermiogenesis [68]. In contrast, adult exposed mice show a decrease in round spermatids, suggesting stage-dependent sensitivity [68]. Histological analysis of testes from rodents chronically exposed to cocaine shows significant degeneration of germ cells, a reduction in seminiferous tubule diameter, and loss of step VII spermatids [57,73,74]. Electron microscopy shows mitochondrial swelling and lipid droplet accumulation in germ cells, suggesting metabolic dysfunction [72]. Importantly, loss of mouse spermatogonia has been confirmed by decreased DAZL-positive cell counts and reduced Dazl levels after cocaine exposure [57]. Among undifferentiated germ cells, DRD1 expression has been specifically detected in spermatogonia located near the basal lamina, raising the possibility that cocaine-induced dopaminergic alterations may directly affect spermatogonial maintenance or differentiation [57].

In summary, these findings reveal that the testis harbors a functional catecholaminergic system that responds to cocaine exposure. This supports the concept of a neuro-testicular axis, whereby systemic catecholaminergic signals—or their disruption—can modulate local testicular physiology. Notably, recent findings indicate that such disruptions can also induce epigenetic reprogramming of male germ cells, potentially contributing to heritable outcomes in the offspring [1,4].

## 4. Germline Epigenetic Alterations and Intergenerational Outcomes of Paternal Cocaine Exposure

The establishment and maintenance of distinct cell identities across tissues, despite the presence of an identical genetic sequence, rely on epigenetic modifications that regulate gene expression [75,76]. These modifications—including DNA methylation, histone post-translational modifications (PTMs), and non-coding RNAs—govern developmental programs and lineage specification. When established in germ cells, they can escape epigenetic reprogramming and persist into the next generation [4,77]. Germline epigenetic modifications are particularly relevant to the transmission of environmentally induced phenotypes, particularly through the paternal lineage. In this section, we examine how cocaine exposure disrupts the epigenetic landscape of germ cells and sperm and explore the resulting effects in the offspring.

### 4.1. Epigenetic Remodeling of Germ Cells and Sperm

Cocaine has emerged as a potent environmental modulator of the epigenome, capable of inducing stable chromatin changes that may be transmitted across generations. While much of the early evidence derives from studies in the nervous system—where chronic cocaine exposure alters DNA methylation and histone PTMs patterns in reward-processing brain regions, more recent work has begun to elucidate similar epigenetic mechanisms in the male germline [78,79]. Cocaine exposure exerts profound effects on the stress-sensitive catecholaminergic system, particularly via activation of DRD1 signaling, which in turn drives downstream epigenetic remodeling. Chronic cocaine use has been shown to increase expression of DNA methyltransferases DNMT3A and DNMT3B in the NAcc, leading to DNA hypermethylation and transcriptional repression [80,81]. Also, cocaine promotes hyperacetylation of histones H3 and H4 in numerous gene promoters and modulates class II HDACs such as HDAC5, whose phosphorylation and nuclear export under cocaine exposure enhances transcription of reward-related genes [81,82]. Together, these data suggest a mechanistic cascade in which cocaine-induced disruption of catecholaminergic signaling—particularly through stress-related dopaminergic pathways—modulates the activity of DNMTs, HDACs, and chromatin-remodeling enzymes. These alterations contribute to the establishment of long-lasting epigenetic states and sustained changes in gene expression in both neural and germline cell populations [81,82].

**Figure 2:**
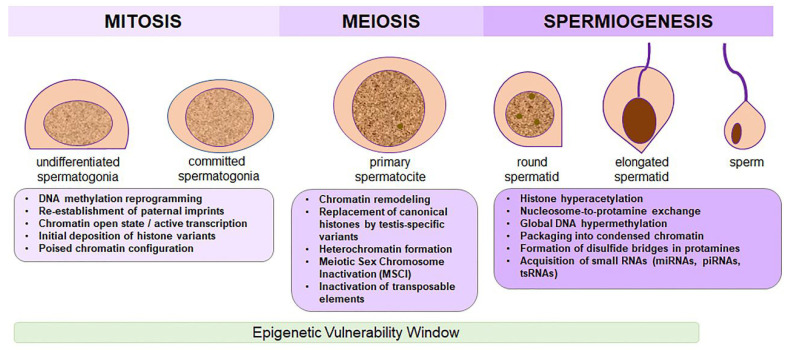
Epigenetic dynamics across spermatogenesis. Schematic representation of the main epigenetic processes occurring during the three phases of spermatogenesis: mitosis (spermatogonial proliferation), meiosis (spermatocyte differentiation), and spermiogenesis (maturation of spermatids into spermatozoa). In the mitotic phase, de novo DNA methylation re-establishes paternal imprints through DNMT3A and DNMT3B, with open chromatin and early deposition of histone variants. Meiosis involves histone replacement (H1t, TH2B), accumulation of repressive histone marks H3K9me3 and H3K27me3, and meiotic sex chromosome inactivation (MSCI). During spermiogenesis, histone hyperacetylation promotes histone eviction, followed by incorporation of transition proteins and protamines. Chromatin compaction and global DNA hypermethylation through DNMT1 and DNMT3A are established. Mature sperm also carry small RNAs—miRNAs, piRNAs, and tsRNAs—acquired during spermatogenesis and epididymal transit. The Figure also highlights a window of epigenetic vulnerability, during which environmental exposure to substances such as cocaine may interfere with the establishment and remodeling of the male germline epigenome, potentially altering the information carried by mature sperm.

During spermatogenesis, germ cells undergo extensive chromatin remodeling; however, certain epigenetic marks can escape global erasure and persist in mature sperm, potentially influencing embryonic development and offspring phenotypes (Figure 2). This occurs during a critical phase known as the epigenetic window, a period of heightened sensitivity during which environmental exposure to substances such as cocaine can induce stable, heritable alterations [83,84,85,86]. Importantly, recent findings indicate that activation of GRs can mediate intergenerational transmission of stress-induced phenotypes via epigenetic mechanisms. Although this has been demonstrated in models using dexamethasone, the underlying pathway is likely relevant in the context of cocaine exposure. It has been shown that pharmacological activation of GR leads to active DNA demethylation in the embryo, establishing persistent chromatin accessibility signatures [87]. Moreover, GR-regulated loci in sperm have been proposed as targets of stress-induced DNA methylation changes, potentially linking paternal experiences to altered transcription and behavior in the offspring [88]. Despite significant advances, the molecular and cellular mechanisms by which epimutations are transmitted at fertilization and retained in the offspring remain poorly understood.

**Figure 3:**
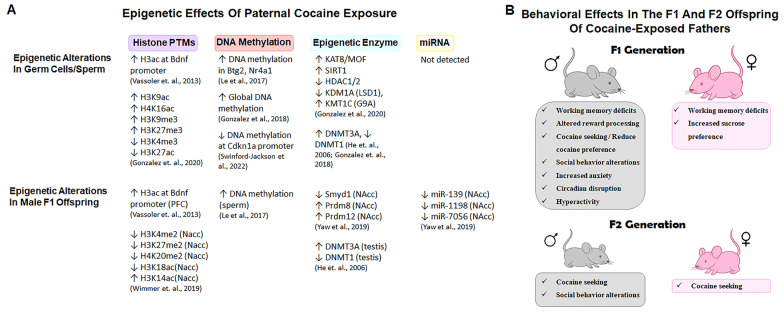
Epigenetic and behavioral effects of paternal cocaine exposure in rodents. (**A**) Epigenetic alterations reported in germ cells/sperm of cocaine-exposed male mice, and in tissues of male offspring, as reported across different experimental studies. Observed changes include post-translational histone modifications (PTMs), DNA methylation, differential expression of epigenetic enzymes, and alterations in microRNA (miRNA) levels. Tissue sources include testis, sperm, prefrontal cortex (PFC), and nucleus accumbens (NAcc) [64,89,91,92,93,94,103,108]. (**B**) Behavioral phenotypes observed in F1 and F2 offspring of cocaine-exposed sires, including sex-specific differences. In male F1 offspring, cocaine exposure resulted in a range of phenotypes, including cognitive, emotional, and social alterations, as well as dysregulation of reward processing and drug-related motivation. Female F1 offspring showed more restricted phenotypes. Effects persisting into the F2 generation were observed primarily in males. Data were extracted from published studies referenced in the main text.

A summary of the main epimutations identified in tubular germ cells and sperm of cocaine-treated rodents across experimental studies is provided in Figure 3A. A pivotal study by Vassoler et al. (2013), showed that cocaine self-administration in male rats results in increased acetylation of H3 at the promoter of the Brain-Derived Neurotrophic Factor (BDNF) in sperm [89]. This epigenetic alteration was associated with elevated BDNF mRNA and protein levels in the medial PFC of the offspring, providing early evidence for functional germline transmission of drug-induced chromatin changes [89]. Subsequent studies by González (2020) showed that cocaine increased H3K9 and H4K16 acetylation—marks of open chromatin—alongside upregulation of the acetyltransferase MOF and deacetylase SIRT1, and downregulation of HDAC1/2 [64]. Cocaine also increased repressive methylation marks H3K9me3 and H3K27me3, and reduced activating marks H3K4me3 and H3K27ac, together with dysregulated expression of the histone methyltransferase G9A and the demethylase LSD1, suggesting a shift toward transcriptional repression [64]. Interestingly, a novel DA-dependent modification, histone dopaminylation (H3Q5dop), has also been identified following cocaine exposure [90]. This modification involves the covalent addition of a DA to glutamine 5 of histone H3, modulating transcriptional programs related to addiction and cocaine-seeking behavior [90]. Although this modification has not yet been reported in germ cells, the presence of a functional dopaminergic system raises the possibility that similar non-canonical histone modifications could occur in germ cells exposed to cocaine.

In addition to histone modifications, cocaine exposure has been associated with altered DNA methylation in male germline. Two independent studies reported reduced expression of DNMT1 and increased expression of DNMT3A in germ cells, consistent with a shift toward de novo methylation and the establishment of repressive marks [64,91]. Increased global DNA methylation was observed in both spermatogenic cells and mature sperm, suggesting stable epigenetic reprogramming [92]. In sperm, cocaine exposure has been associated with widespread methylation changes across regulatory regions. Le et al. (2017) identified hypermethylation at synaptic genes [93], while Swinford-Jackson et al. (2022) reported hypomethylation in two regions of the Cdkn1a promoter [94], indicating that cocaine may impact different genomic regions via distinct methylation dynamics.

As another layer of epigenetic regulation, small non-coding RNAs—including miRNAs, piRNAs, and tsRNAs—are essential in germline epigenetic regulation [95]. They participate in post-transcriptional gene silencing, transposon repression, and chromatin organization, and are selectively retained in mature sperm, functioning as vectors of environmentally induced information [95]. However, studies addressing their modulation by cocaine remain limited. Swinford-Jackson et al. (2022) analyzed miRNA profiles in sperm from cocaine-exposed rats and found no consistent changes compared to controls, suggesting possible resistance of sperm miRNA content under the tested conditions [94]. Still, given the established role of sncRNAs in intergenerational inheritance in other models [96], future studies should examine other RNA classes, exposure paradigms, and developmental windows. Notably, sperm-borne mRNAs have been shown to contribute to chromatin remodeling and zygotic genome activation [97], and may represent additional targets of cocaine-induced disruption.

### 4.2. From Sperm to Offspring: Intergenerational Effects of Paternal Cocaine Use

The transmission of environmentally induced epigenetic information across generations is commonly categorized as either intergenerational or transgenerational inheritance. In paternal exposure models, intergenerational inheritance refers to phenotypic or molecular alterations observed in the F1 generation that result from direct modifications in germ cells or in mature sperm [98,99,100,101,102]. In contrast, transgenerational inheritance implies persistence of these alterations in F2 generation or beyond—in generations that were not directly exposed—indicating that certain epigenetic marks can escape the reprogramming events that occur during gametogenesis and early embryonic development [100,101]. Pioneering studies by Skinner and colleagues demonstrated that transient exposure to the endocrine disruptor vinclozolin during the gonadal sex determination period induces heritable DNA methylation changes in the male germline, with disease susceptibility across generations [102]. These findings laid the foundation for current models of environmentally induced epigenetic inheritance. Among toxicological stressors, cocaine has emerged as a relevant candidate for epigenetic transmission, given its capacity to induce stable epimutations in the male germline.

Multiple factors have been shown to influence the outcomes of paternal cocaine exposure, including the dose, route, and duration of administration; timing of mating; and sex of the offspring. Notably, self-administration paradigms differ from fixed-dose injection models or oral exposure protocols in the physiological and motivational pathways they engage, potentially influencing the nature and extent of epigenetic signatures transmitted to the next generation (Figure 3A). Male offspring generally exhibit greater susceptibility to cocaine-related phenotypes, suggesting sex-specific differences in behavioral outcomes following paternal exposure (Figure 3B). A detailed summary of the main studies reporting epigenetic alterations and phenotypic outcomes in offspring following paternal cocaine exposure is provided in Supplementary Table S1.

Self-administration paradigms have provided unique insights into intergenerational transmission. Early work by Vassoler et al. (2013) showed that cocaine self-administration in sires confers resistance to cocaine in male F1 offspring [89]. This phenotype was associated with increased acetylation of H3K9K14ac2 at the Bdnf promoter in sperm of cocaine-exposed sires [89]. Subsequent studies also reported reduced cocaine motivation in F1 males [94], while others found increased intake and drug-seeking behavior, accompanied by alterations in synaptic plasticity-related genes and memory impairments [93,103]. Additionally, Huang et al. (2024) showed that drug-seeking behavior—not passive cocaine exposure in yoked controls—reduced the expression of a GABA-A receptor subunit in the VTA area of male F1 offspring, an alteration causally linked to enhanced motivation [104]. These findings highlight a key distinction: the motivational context in which cocaine is consumed appears to shape the epigenetic landscape inherited by male F1. Offspring of sires that actively seek the drug display distinct molecular and behavioral outcomes compared to those whose fathers received passive exposure, emphasizing the relevance of active drug-seeking in the generation of heritable epigenetic effects.

Fixed-dose paradigms have been linked to anxiety-like behavior and hyperactivity in male F1 rats [37,105,106]. White et al. (2016) reported increased expression of corticotropin-releasing factor receptor 2 (CRF-R2) in the hippocampus of F1 males, suggesting dysregulation of the HPA axis as a molecular contributor to the anxiogenic phenotype [107]. Other findings include increased depression-like behavior and hyperactivity in F1 males, while females show either no changes or enhanced preference for natural rewards such as sucrose [107]. The results for the cognitive outcome are variable: some studies report no alterations in spatial memory [104], whereas others describe working memory impairments along with reductions in hippocampal LTP (long-term potentiation) and D-serine levels—key molecular components of synaptic plasticity [78]. He et al. (2006) found working memory deficits in both sexes and observed reduced expression of Dnmt1 and Dnmt3a in the testes of cocaine-exposed sires [91]. Finally, oral exposure protocols show that F1 males exhibit reduced cocaine preference and transcriptomic alterations in the NAcc, including downregulation of genes related to extracellular matrix remodeling and immune signaling [108]. In contrast, F1 females display heightened sucrose preference but no cocaine-related changes [108].

Beyond differences in exposure paradigms, additional studies have explored whether these paternal effects extend to the F2 generation. Wimmer et al. (2019) reported that histone modifications observed in the NAcc of F1 males-such as decreased H3K4me/H3K27me2/H4K20me2/H3K18ac, and increased H3K14ac-were not detected in F2, suggesting partial reversion [103]. Le et al. (2017) demonstrated that 475 CpG sites differentially methylated in the sperm of cocaine-exposed sires were also altered in F1 sperm, indicating inheritance of methylation patterns [93]. Moreover, these changes were enriched in promoters of genes related to neurodevelopment and synaptic signaling, and male F2 offspring of addicted sires displayed increased cocaine intake and motivation [93]. In addition, Yaw et al. (2019) observed that transcriptomic and behavioral changes present in F1 were absent in F2, accompanied by normalization of cocaine preference [108]. They also reported altered expression of miRNAs and histone methyltransferases Smyd1, Prdm8, and Prdm12 in the NAcc of F1 males, supporting an epigenetic basis for the observed phenotypes [108]. Other behavioral traits limited to F1 include circadian rhythm disruption [109], increased stress reactivity [105], and social behavior impairments [110].

While rodent studies offer strong support for epigenetic inheritance following paternal cocaine exposure, human data remain limited. A few epidemiological studies suggest associations with increased risk of congenital abnormalities, infant mortality, or childhood cancers [111], but these findings are correlational, often based on retrospective maternal reporting, and lack molecular analyses of male germ cells. To date, no human studies have directly examined epigenetic alterations in sperm following paternal cocaine use, underscoring the need for translational research.

## 5. Conclusions and Future Perspectives

The evidence reviewed here showed the profound impact of cocaine on male reproductive physiology, from hormonal imbalance and testicular cell damage to epigenetic reprogramming of the germline. Among these effects, epigenetic alterations are of particular concern because they are not confined to the exposed individual but can be transmitted to the offspring, where they manifest as changes in behavior, gene expression, and chromatin structure. Together, the evidence points to the male germline as both a target and a vehicle of environmentally induced epimutations. Despite growing interest in this field, several critical questions remain. Most of the previous studies have focused on either the testis or the offspring, but few have simultaneously examined the entire cascade—from germ cell alterations to intergenerational outcomes—within the same experimental framework. The integration of data across timepoints and tissues is essential to clarify the mechanisms that mediate epigenetic inheritance.

A particularly novel aspect highlighted in this review is the potential involvement of a local catecholaminergic system in the testis as a mediator of cocaine-induced germline effects. The presence of receptors, transporters, and catecholamine-synthesizing enzymes in testicular cells suggests a functional pathway that may link systemic drug exposure to local epigenetic changes. Although this mechanism remains understudied, it offers a promising direction for future research. Finally, while rodent models have been instrumental in establishing causal links between paternal drug exposure and heritable phenotypes, translational studies in humans are urgently needed. The development of high-resolution molecular tools to analyze human sperm epigenomes—alongside prospective epidemiological studies—may help uncover the relevance of these findings for human health and reproduction. Understanding how environmental exposure to substances such as cocaine influence the germline and shape the next generation will be key to addressing the long-term consequences of substance use, with implications for public health, reproductive medicine, and epigenetic theory.

## Data Availability

No new data were created or analyzed in this study.

## Supplementary Materials

The following supporting information can be downloaded at https://www.mdpi.com/article/10.3390/biology14081072/s1, Table S1: Paternal Cocaine Exposure Studies: Experimental Design, Epigenetic Alterations, and Offspring Outcomes.
